# Commentary: Duration-dependent effects of the BDNF Val66Met polymorphism on anodal tDCS induced motor cortex plasticity in older adults: a group and individual perspective

**DOI:** 10.3389/fnagi.2015.00183

**Published:** 2015-09-23

**Authors:** Anna Shpektor, David Bartrés-Faz, Matteo Feurra

**Affiliations:** ^1^School of Psychology, Centre for Cognition and Decision Making, National Research University Higher School of EconomicsMoscow, Russia; ^2^Department of Psychiatry and Clinical Psychobiology, University of BarcelonaBarcelona, Spain; ^3^Unit of Neurology and Clinical Neurophysiology, Brain Investigation and Neuromodulation laboratory (Si-BIN Lab), Department of Medicine, Surgery and Neuroscience, Azienda Ospedaliera Universitaria of SienaSiena, Italy

**Keywords:** BDNF, tDCS, synaptic plasticity, primary motor cortex, aging

The Brain Derived Neurotrophic Factor (BDNF) is a neurotrophic protein that is strongly expressed in the Central Nervous System (CNS) and supports neuronal growth and survival (Conner et al., 1997). BDNF activates two neurotrophin receptors: Tropomyosin receptor kinase B (TrkB), that is necessary for hippocampal long-term potentiation (LTP) and associative learning, and Low-Affinity Nerve Growth Factor Receptor (P75^NTR^), that is required for hippocampal long-term depression (LTD; Patterson et al., 1996; Gruart et al., 2007; Chapleau and Pozzo-Miller, 2012). Val66Met is a common single nucleotide polymorphism (SNP) placed in the human BDNF gene that leads to an amino-acid substitution of valine to methionine at codon 66 and cause a 18–30% decrease of BDNF neurotrophin secretion (Egan et al., 2003; Chen et al., 2006). In Met-carriers, the reduction of BDNF secretion results in variation of cortical morphology (Pezawas et al., 2004). This affects performance of memory and learning tasks (Egan et al., 2003; Hariri et al., 2003) and it may cause neuropsychological disorders (Lu et al., 2013; Moreira et al., 2015; van der Kolk et al., 2015). On this vein, it has been shown that Val66Met polymorphism play a complex role both for plasticity changes and recovery processes in after-stroke patients (Di Lazzaro et al., 2015; Di Pino et al., 2015).

The Val66Met polymorphism also alters sensorimotor system processes. McHughen et al. (2010) showed that during a simple right index finger movement task, Val/Met carriers exhibited reduced activation volumes on different brain regions (including motor cortex, premotor cortex, supplementary motor area) with respect to Val/Val carriers. Interestingly, after training, Val/Val showed a greater activation volume expansion whereas Val/Met carriers a greater activation volume reduction. Moreover, inside a driving-based motor learning task, subjects with polymorphism benefitted less from training. All together, these findings highlight that differences in volume activation, accordingly to the polymorphism, increase with presence of training.

Recently, it has been shown that Transcranial Direct Current Stimulation (tDCS), might be an optimal tool to investigate the role of BDNF polymorphism in activity-dependent plasticity (Fritsch et al., 2010). In the human motor system, tDCS induces long-lasting and polarity-specific changes in the excitability of the motor cortex (Nitsche and Paulus, 2000). Anodal tDCS induces facilitatory effect by a membrane depolarization and by an increase in the excitability of corticospinal axons (Di Lazzaro et al., 2013), while cathodal tDCS induces inhibitory effects by a membrane hyperpolarization (Wagner et al., 2007). Animal models showed that tDCS modify thalamocortical synapses at presynaptic sites by changes in the membrane potential of cortical neurons (Márquez-Ruiz et al., 2012).

As discussed above, BDNF protein partly modulates LTP and LTD; interestingly it also modulates the effect of tDCS in both humans and animals. In mice, Fritsch and collaborators (Fritsch et al., 2010) showed that anodal tDCS coupled with repetitive low-frequency synaptic activation (LFS) induced a long-lasting synaptic potentiation (DCS-LTP). BDNF was a key modulator of this process: its secretion was enhanced by coupled tDCS-LFS and tDCS did not induce LTP in the absence of BDNF secretion (BDNF and TrkB mutant mice). At the same time, anodal tDCS over the primary motor cortex (M1) induced minor effects on the group with a reduced BDNF expression (Val/Met carriers) during a pinch-force task. Taken together, these results showed that tDCS might improve motor skills by inducing synaptic plasticity, which requires BDNF secretion in both humans and mice (Fritsch et al., 2010).

In a recent paper in *Frontiers in Aging Neuroscience*, Puri and collaborators (Puri et al., 2015), addressed the question about BDNF influence on tDCS effects in aging. They applied anodal tDCS for 10 and 20 min by stimulating left M1 on Val/Val and Val/Met subjects at rest. By using single pulse TMS, corticospinal excitability was measured before and each 5 min after tDCS offset. Results showed group- and time-dependent effects: Met carriers showed higher increase of M1 cortical excitability compared to Val/Val carriers. This effect was present only after 20 min of stimulation. Despite a previous tDCS study highlighted brain stimulation effects to be more pronounced on a Val/Met group (Antal et al., 2010), here authors showed the first evidence of the Val66Met SNP influence on M1 cortical excitability when tDCS was delivered at rest, as reflected by a significant between-groups interaction. These findings may be controversial: based on the previous literature, tDCS effects at rest did not differ between Val/Val and Val/Met carriers (Cheeran et al., 2008; Di Lazzaro et al., 2012; Fujiyama et al., 2014). Instead, influence of Val66Met polymorphism becomes more pronounced during task performance (Fritsch et al., 2010; McHughen et al., 2010). This might be due to the activity-dependence of BDNF secretion (Egan et al., 2003). The study by Puri et al. (2015) is also the first one that pointed out the crucial role of interaction between Val66Met SNP and duration of tDCS-M1 at rest: differences in cortical excitability occurred after 20 min of stimulation, but not after 10 min. However, these results do not fit with previous studies which showed no differences by tDCS at rest, when stimulation was applied for 10, 20, and 30 min (Cheeran et al., 2008; Di Lazzaro et al., 2012; Fujiyama et al., 2014). Results by Puri et al. (2015), may have different interpretations.

One possibility is that the two groups were unbalanced in terms of number of subjects: the sample size of Val/Val carriers was three times higher than Val/Met carriers. On one hand, this allowed authors to be blinded to the genotype during the experiment. On the other hand, since tDCS affected only the smaller group (Met-carriers), unbalanced analysis might have affected statistical sensitivity (Hector et al., 2010). Another possibility is that BDNF protein could have affected differently motor control processes in elder population, as was shown that its influence on memory functions changes during the lifespan (Kennedy et al., 2015; Papenberg et al., 2015). In summary the study by Puri and collaborators showed an interesting effect of Val66Met SNP in aging. Results were also stable as shown by less inter-individual variability inside the Met group which showed a more robust tDCS after-effect. The fact that 77% of Met carriers but only 35% of Val carriers showed the expected facilitation in both sessions could be used to stratify sample selection in aging studies where more homogeneous responses to Non Invasive Brain Stimulation are required. Finally, a further study with larger and balanced group of participants is needed. It will be interesting to have a control group of younger adults to understand the role of aging in the BDNF influence on motor plasticity at rest.

## Conflict of interest statement

The authors declare that the research was conducted in the absence of any commercial or financial relationships that could be construed as a potential conflict of interest.
